# Transportal Tibiotalocalcaneal Nail Ankle Arthrodesis: A Systematic Review of Initial Series

**DOI:** 10.1177/24730114231156422

**Published:** 2023-02-28

**Authors:** Darius Luke Lameire, Hassaan Abdel Khalik, Christopher Del Balso, Timothy Daniels, Mansur Halai

**Affiliations:** 1 Division of Orthopaedic Surgery, Department of Surgery, University of Toronto, Toronto, ON, Canada; 2 Division of Orthopaedic Surgery, Department of Surgery, McMaster University, Hamilton, ON, Canada; 3 Department of Orthopaedic Surgery, London Health Sciences Centre, Western University, London, ON, Canada; 4 Unity Health Toronto–St Michael’s Hospital, Toronto, ON, Canada

**Keywords:** AOFAS: American Orthopaedic Foot & Ankle Society, avascular necrosis, confidence interval, diabetes mellitus, Embase, Excerpta Medica Database, Medline, Medical Literature Analysis and Retrieval System Online, MINORS, Methodological Index for Non-randomized Studies, not reported, PRISMA, Preferred Reporting Items for Systematic Reviews and Meta-Analysis, TTC, tibiotalocalcaneal, VAS-FA, Visual Analog Scale Score Foot and Ankle

## Abstract

**Background:**

There is currently a scarcity of information and consensus for transportal (arthroscopic or fluoroscopic) joint preparation during tibiotalocalcaneal (TTC) fusion, and therefore this review aims to summarize the available techniques and to evaluate the outcomes after this procedure.

**Methods:**

A systematic electronic search of MEDLINE, EMBASE, and Web of Science was performed for all English-language studies published from their inception to April 4, 2022. All articles addressing arthroscopy in TTC nailing were eligible for inclusion. The PRISMA Checklist guided the reporting and data abstraction. Descriptive statistics are presented.

**Result:**

A total of 5 studies with 65 patients were included for analysis. All studies used arthroscopic portals for tibiotalar and subtalar joint preparation (in 4 studies) prior to TTC nailing, with 4 studies using an arthroscope and 1 study using fluoroscopy. The overall major complication rate was 13.8%; however, there was only 1 instance of deep wound infection (1.5%) and 4 instances of surgical site infections (6.2%). Full fusion was achieved in 86% of patients with an average time to fusion of 12.9 weeks. The mean American Orthopaedic Foot & Ankle Society (AOFAS) ankle-hindfoot score preoperatively was 34.0 and postoperatively was 70.5.

**Conclusion:**

Although limited by the number of studies, transportal joint preparation during TTC nail ankle fusion is associated with good rates of complications and successful fusion.

**Level of Evidence:**

Level III, systematic review of Level III-IV studies.

## Introduction

Tibiotalocalcaneal (TTC) fusion is a surgical procedure aimed to achieve restriction of movement of the TTC joint and to relieve pain caused by motion in the ankle. The benefits of intramedullary nailing compared with other methods of fusion include more load sharing during healing and less soft tissue injury, potentially leading to earlier weightbearing.^11,23^ TTC fusion is indicated for the treatment of avascular necrosis (AVN) of the talus, severe osteoarthritis of both the tibiotalar and subtalar joint, failed total ankle arthroplasty with ipsilateral arthritis of the subtalar joint, or associated avascular necrosis of the talus. It has also been proven to be beneficial in managing neuromuscular diseases, trauma, clubfoot, Charcot arthropathy, pseudoarthrosis, and congenital deformities.^1,5,8,21^

Although open arthrodesis of the ankle to prepare the tibiotalar and subtalar joints for TTC fusion has been the gold standard, it requires wide incisions that can cause problems with wound healing particularly in patients with coagulopathy, steroid use, peripheral vascular disease, rheumatoid arthritis, and diabetes.^22^ Transportal (arthroscopic and/or fluoroscopic) TTC fusion is an adjunct that can reduce the invasiveness and soft tissue damage when preparing the joint for fusion and has been shown to have promising results.^2,17^ The proposed benefits also include reduced postoperative pain, swelling, and wound complications, as well as a reduced hospital stay.^2,9^

Although there was a recent study evaluating TTC arthrodesis, it was limited because there was only 1 primary research article published at that time analyzing the outcomes of arthroscopy-assisted minimally invasive TTC nailing, and they were unable to draw strong conclusions regarding this surgical technique.^9^ As such, this is the first systematic review to assess the benefits of minimally invasive joint preparation in TTC arthrodesis while also providing a needed update on the literature describing the technique, outcomes, and complications with transportal joint preparation. As transportal TTC nailing is still a relatively new and technically demanding procedure, there is no consensus on the surgical steps including, but not limited to, optimal patient positioning, arthroscopic portals, and the use of intraoperative fluoroscopy.

Given this lack of agreement, the aim of this systematic review is to summarize the procedural options for minimally invasive transportal tibiotalar and subtalar joint preparation for TTC nail arthrodesis, as well as to determine the clinical, functional, and complication outcomes following this innovative surgical technique.

## Methods

This systematic review focused on the technique, outcomes, and complications after transportal joint preparation in TTC arthrodesis. This review followed the guidelines and algorithm of the Preferred Reporting Items for Systematic Reviews and Meta-Analysis (PRISMA).^16^

### Comprehensive Search Strategy

A systematic search of 3 databases (the Medical Literature Analysis and Retrieval System Online (Medline), the Excerpta Medica Database (Embase), and Web of Science) and manual search of references was performed through April 4, 2022, by 2 reviewers (DLL and HAK) for literature related to arthroscopy in the context of TTC nail ankle fusion. The search terms included tibiotalocalcan*, *arthrodesis*, *fusion*, and *arthroscopy*. The complete search strategies can be found in Appendix 1. The inclusion criteria for this review were (1) TTC fusion using an intramedullary rod, (2) arthroscopy or the use of minimally invasive arthroscopic portals to prepare the tibiotalar and/or subtalar joint, (3) studies available in English, (4) studies including at least 5 patients, (5) outcomes data provided, (6) 18 years and older, and (7) all levels of evidence. Exclusion criteria consisted of (1) open TTC nail fusion, (2) cadaveric studies, (3) biomechanical studies, (4) no follow-up/outcomes data reported, (5) pediatric population, and (6) systematic reviews. Both arthroscopic (using an arthroscope in particular) and arthroscopic portal (creating portals and using other adjuncts such as fluoroscopy and curettes) joint preparation prior to TTC nailing were included in this study because of the similar small incisions and minimal soft tissue disruption associated with the procedure. For the purposes of this review, both minimally invasive techniques using an arthroscope, or arthroscopic portals with fluoroscopy will be referred to as “transportal TTC nailing.”

### Study Screening

The titles and abstracts of the studies were independently assessed by 2 authors (D.L.L. and H.A.K.) using the above inclusion and exclusion criteria. To prevent any premature exclusion, all disagreements and studies with insufficient data were advanced to the full-text review stage. The third author (M.H.) resolved any disagreements. The level of agreement between reviewers was assessed by calculating a Kappa (κ) score.^14^ The quality of included studies were assessed using the Methodological Index for Non-randomized Studies (MINORS) and the scores were averaged (Table 1).^24^

**Table 1.** Study Demographics.^a^

Author	Patients, n	Ankles, n	Female, n (%)	Age, Years (Range)	Mean Follow-up, mo (Range)	Indication for Surgery	MINORS Score
Baumbach (2019)^2^	15	15	1 (6.7)	56 (NR)	12 (NR)	Charcot neuro-osteoarthropathy: 8 Combined posttraumatic osteoarthritis: 4 Equinus following Chopart amputation: 2 Neurogenic equinus/clubfoot: 1	17.5
Biz (2016)^3^	28	28	6 (21)	52 (35-80)	25 (6-40)	Combined posttraumatic osteoarthritis: 18 Primary osteoarthritis: 6 Charcot neuro-osteoarthropathy: 2 Neurogenic equinus/clubfoot: 2	13
Guerra Álvarez et al^11^	7	7	1 (14)	65 (47-76)	NR (NR)	Primary osteoarthritis: 3 Combined posttraumatic osteoarthritis: 3 Neurogenic equinus/clubfoot: 1	11
Mencière et al^15^	6	6	3 (50)	58 (40-74)	17 (12-22)	Neurogenic equinus/clubfoot: 5 Charcot neuro-osteoarthropathy: 1	10
Sekiya et al^22^	8	9	6 (75)	52 (22-72)	41 (16-72)	Rheumatoid arthritis: 6 Drop foot: 1 Primary osteoarthritis: 1	11.5
Mean ± SD (95% CI)			26.4% ± 21.6% (7.4%-45.3%)	54.9 ± 4.1 (51.3-58.5)	23.3 ± 9.3 (14.1-32.4)		

Abbreviation: NR, not reported.

a All the studies and their included patient characteristics.

### Data Abstraction

The data from each study was abstracted into predetermined tables using Google Sheets by 2 reviewers (D.L.L. and H.A.K.) and further reviewed by the third author (M.H.). The following data were abstracted from the studies if available: study characteristics (author, publication year, journal, level of evidence, study design, etc), number of patients, participants characteristics (i.e., age, sex, etc), follow-up length, details of the treatment performed (arthroscopic portals, TTC nail, etc), subjective outcomes (the American Orthopaedic Foot & Ankle Society [AOFAS] ankle-hindfoot score, visual analog scale for pain, etc) pain scale, and complications. Descriptions of the procedures, arthroscopy portals, patient positioning, and instruments used were summarized.

### Primary Outcomes

The primary outcomes of this study were complications, percentage of patients achieving bony fusion, and time to bony fusion. Complications were divided into major and minor complications. Major complications consisted of symptomatic nonunion, asymptomatic nonunion, tibiotalar pseudoarthrosis, intraoperative fracture, and deep wound infection. Minor complications consisted of discomfort of the osteosynthesis, exostosis, screw loosening, nonclinical subtalar pseudoarthrosis, and superficial surgical site infection. Bony fusion was defined as time to achieve bony union as described by each article, respectively, or fusion of both the tibiotalar and subtalar joint.

### Secondary Outcomes

The secondary outcomes consisted of patient and clinical outcomes. The clinical and patient-reported outcomes consisted of AOFAS ankle-hindfoot score/Kitoaka Score and the visual analog scale score foot and ankle (VAS-FA).^13,26^ Although the AOFAS score is not validated and although it is no longer recommended by the AOFAS, it was the primary measurement used by the studies that qualified for this review. The VAS-FA score is a validated questionnaire consisting of 20 subjective questions assessing pain, function, and other complaints. A score of zero corresponds to an ankle and/or foot with debilitating pain, impaired function, and one that is extremely limiting and 100 corresponds to a completely pain-free, fully functional, nonlimiting ankle/foot.^20^

### Statistical Analysis

Where possible, descriptive statistics were derived using R (R Studio, Boston, MA). These included weighted means and SDs, as well as 95% CIs. Weighted means were calculated by comparing the value of the outcome of interest from each study (ie, AOFAS score) and calculated the relative weighting of that score based on the number of patients included in the study. This was completed for all studies and then added together to determine the overall weighted average. Otherwise, outcomes were presented in narrative summary fashion.

## Results

**Figure 1.**
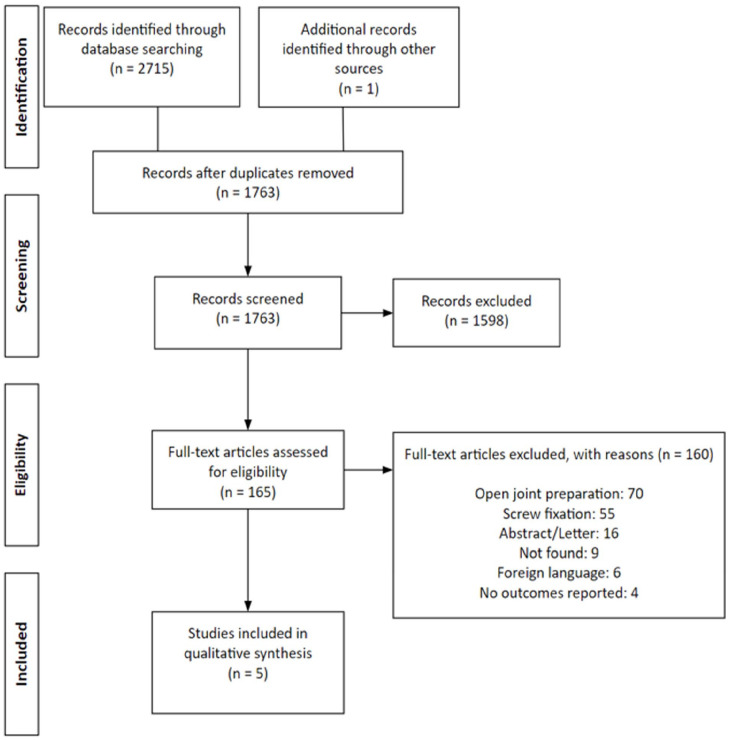
PRISMA.

A total of 2715 articles were identified initially and 1763 articles remained after removal of duplicates, of which 5 articles were included for this review (Figure 1).^2,3,11,15,23^ There was substantial agreement obtained for the title and abstract screening (κ = 0.769; 95% CI, 0.712-0.826) and almost perfect agreement for full-text screening (κ = 0.886; 95% CI, 0.664-01.000). There were 4 Level IV evidence case series,^3,11,15,23^ and 1 Level III evidence retrospective cohort study^2^ included in this systematic review. The average MINORS assessment score for the comparative study was 17.5 and the average MINORS score for all noncomparative studies was 11.4 (Table 1).

### Patient Demographics

The included studies consisted of 65 patients with a mean age of 59.4 ± 4.1 years old. The average percentage of female patients was 26.4% ± 21.6%, and the average follow-up period was 23.3 ± 9.3 months. Of the included patients, 25 patients underwent TTC fusion for combined posttraumatic osteoarthritis, 11 for Charcot neuro-osteoarthropathy, 10 for primary osteoarthritis, 9 for neurogenic equinus/clubfoot, 6 for rheumatoid arthritis, 2 for equinus formation following Chopart amputation, and 1 for drop foot (Table 1).

### Patient Setup

Three studies^2,3,23^ (52 ankles, 80%) positioned the patients in supine, and 2 studies^11,15^ (13 ankles) positioned the patients in prone. Two studies^3,11^ used spinal anesthesia (35 patients), and the other 3 studies^2,3,23^ did not report which anesthesia they used. One study^11^ (7 patients) used a tourniquet, 1 study^3^ reported not using a tourniquet (28 patients), and 3 studies^2,15,23^ did not report on tourniquet use. The median time of ischemia for the study that used a tourniquet was 2 hours. The one study^3^ that reported specifically not using a tourniquet (28 patients) had an average length of surgery of 80.16 ± 8.12 minutes. No studies reported on the use of irrigation. Full patient setup details can be found in Appendix 2.

### Joint Preparation and Insertion of the Intramedullary Nail

Four studies used an anterolateral portal, 3 studies used a posterolateral portal, 3 studies used an anteromedial portal, 2 studies used a posteromedial portal, and 1 study used a lateral portal to the subtalar joint through the sinus tarsi. The posterolateral and posteromedial portals were those previously described by van Dijk et al.^25^ Four studies (36 patients; 56.3%) specifically used an arthroscope,^2,11,15,23^ and 1 study (28 patients) reported using minimally invasive arthroscopic portals with the use of fluoroscopy to aid joint preparation.^3^ All studies prepared the joint by removing cartilage and subchondral bone down to bleeding bone using a combination of shavers, burrs, curettes, etc. The full description of the steps for joint preparation and arthroscopes used from each study can be found in Table 2.

**Table 2.** Joint Preparation.^a^

Author	Achilles Tenotomy	Portals	Arthroscope	Key Steps
Baumbach et al^2^	Yes (percutaneous)	1. Anteromedial and anterolateral portals (to approach the tibiotalar joint) 2. Anterolateral and posterolateral portals (to approach the subtalar joint)	NR	1. Percutaneous Achilles tendon tenotomy 2. Insertion of portals 3. Articular surfaces debrided until subchondral bone was exposed 4. After proper hindfoot alignment and surface fit were insured, subchondral bone marrow was perforated 5. Hindfoot was temporarily fixed using 2.0 K-wires
Biz et al^3^	NR	1. Anterolateral subtalar arthroscopy portal—a 1- to 1.2-cm vertical skin incision using a No. 11 scalpel for anterolateral subtalar portal first 2. Posteromedial and posterolateral (Van Dijk)	No	1. Subtalar preparation using surgical chisels first, followed by small osteotomes and a motorized drill with a 5-mm burr under fluoroscopic guidance. Down to bleeding and well-vascularized bone. 2. Same preparation steps for the articular surfaces of the ankle through the anterolateral and anteromedial portals. The articular surface of the talar dome, tibia plafond, and part of the posterior ankle compartment were prepared down to subchondral bone.
Guerra Álvarez et al^11^	NR	1. Posterolateral portal (tibiotalar)—1 cm proximal to the distal tip of the external malleolus, entering the tibiotalar joint toward the second metatarsal 2. Posteromedial portal (subtalar) is created at the same height as the other portal under direct observation	1. 4.5-mm arthroscope with 30-degree angle	1. Using a synoviotome, the ligament of Rouvière and Canela, the intermalleolar ligament, and the posteroinferior tibiofibular ligament is resected. The flexor hallucis longus was used as a reference to determine the limitation in resection of the medial part of the joint. 2. Using a 4-mm high-speed reamer, the tibiotalar and posterior subtalar joint surfaces are reamed 1-2 mm to bleeding. 3. A fibula osteotomy is performed to improve tibiotalar compression
Mencière et al^15^	Yes (percutaneous)	1. Posterolateral and posteromedial portals	NR	1. Percutaneous Achilles tendon tenotomy 2. Visualization of the posterior tibiotalar and talocalcaneal interlines using a radiofrequency system (VAPR, DePuy Orthopaedics Inc, Warsaw, IN) and a bone shaver 3. The tendon of flexor hallucis longus (FHL) was identified, as it provided the boundary of posteromedial debridement. FHL was cut in the case of interphalangeal joint flexion contractures. 4. Joints were heightened using a shaver and curettes in order to expose subchondral bone.
Sekiya et al^22^	NR	1. Anteromedial and anterolateral portals 2. 1-cm lateral sided incision to access the subtalar joint through the sinus tarsi	1. 4-mm 30-degree oblique arthroscope 2. 2.7-mm 30-degree oblique arthroscope	1. Synovectomy with motorized shaver. Straight and angled curettes were used to remove degenerated cartilage from the tibia, talus, and fibula. 2. A 4.5-mm abrader burr and angled curette were used to remove subchondral bone and expose viable cancellous bone. 3. At the medial and lateral gutters, a small curved curette removed subchondral bone and cartilage under visualization with the 2.7-mm arthroscope. 4. A 1-cm skin incision was added at the lateral side of the foot to access the subtalar joint through the sinus tarsi. 5. An angled curette was introduced into the posterior talocalcaneal joint under fluoroscopic guidance and the cartilage of the joint was thoroughly curetted and removed.

Abbreviation: NR, not reported.

a The steps for joint preparation as described in each article along with description of the portals used.

Five different TTC intramedullary nail implants were reported being used. All intramedullary nails were inserted through a plantar incision. Full details of the steps for inserting the intramedullary nail and the position of fusion, as per each study, can be found in Appendix 3.

### Postoperative Protocol

Four studies reported placing patients in a splint ranging from 1 week to 8 weeks. Patients were transitioned to full weightbearing at varying times ranging from 2 to 12 weeks (Appendix 4). One study^3^ (28 patients) reported using nadroparin calcium for thromboembolic prophylaxis after surgery until complete weightbearing.

### Complications

There were a total of 25 complications reported among a total of 65 ankles (38.5%). There were 9 major complications (13.8%) and 16 minor complications (24.6%) (Table 3). The most common major complication was symptomatic nonunion (7.8%), and the most common minor complication was discomfort of the osteosynthesis (9.2%).

**Table 3.** Complications.^a^

	Baumbach et al^2^	Biz et al^3^	Guerra Álvarez et al^11^	Mencière et al^15^	Sekiya et al^22^	All
Ankles, n	15	28	7	6	9	65
Major complications, n						
Symptomatic nonunion	4				1	5
Tibiotalar pseudoarthrosis			1			1
Intraoperative fracture				1		1
Deep wound infection			1			1
Asymptomatic nonunion	1					1
Subtotal	5	0	2	1	1	9
Complication rate, %	33.3	0.0	28.6	16.7	11.1	13.8 ± 14.0 (95% CI 1.6-26.1)
Minor complications						
Discomfort of osteosynthesis (posterior calcaneal screw), n		1	4	1		6
Exostosis, n		3				3
SSI not requiring operative management, n	3					3
Nonclinical subtalar pseudoarthrosis, n			2			2
SSI requiring debridement and antibiotics, n			1			1
Screw loosening, n	1					1
Subtotal, n	4	4	7	1	0	16
Complication rate, %	26.7	14.3	100	16.7	0.0	24.6 ± 27.4 (95% CI 0.6-48.6)
Total complications, n	9	4	9	2	1	25
Overall complication rate, %	60.0	14.4	128.6	33.3	11.1	38.5 ± 37.2 (95% CI 0-57.2)

Abbreviation: SSI, surgical site infection.

a Major and minor complications broken down by each specific type of complication.

### Bone Fusion

All 5 studies reported bone fusion rates with a weighted average of 86% ± 17% of patients achieving full tibiotalar and subtalar ankle fusion. The average percentage of bone fusion ranged from 57.1% to 100%. Four studies reported average time to full ankle fusion or tibiotalar fusion with an average of 12.9 ± 2.0 weeks (range: 10-14.85 weeks) (Table 4).

**Table 4.** Fusion Outcomes.^a^

Author	Number of Ankles	Ankles With Full Bony Fusion, n	% of Patients With Full Ankle Fusion	Time to Bony/Tibiotalar Fusion, wk, mean ± SD (range)
Baumbach et al^2^	15	10	66.7	11 ± 4
Biz et al^3^	28	28	100	14.9 ± 4.1 (8-56)
Guerra Álvarez et al^11^	7	4	57.1	10
Mencière et al^15^	6	6	100	11.7 (10-12)
Sekiya et al^22^	9	8	88.9	NR
		Means	86 ± 17 (95% CI 71-100)	12.9 ± 2.0 (95% CI 10.9-14.9)

Abbreviation: NR, not reported.

a Characteristics and timing of bony fusion as defined by each article.

### Patient/Clinical Outcomes

**Figure 2.**
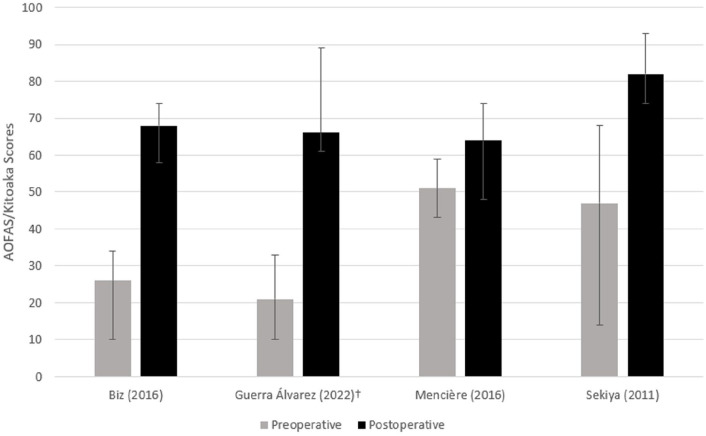
AOFAS/Kitoaka Scores. The preoperative and postoperative AOFAS/Kitoaka scores if reported in each study. Error bars denote minimum and maximum values. ^†^Median values. AOFAS, American Orthopaedic Foot & Ankle Society ankle-hindfoot score.

There were 4 studies that reported preoperative and postoperative AOFAS/Kitoaka Ankle and Foot scores.^2,3,11,15^ Three studies^3,15,23^ reported preoperative means with a weighted average of 34.0 ± 10.8, and 1 study reported a preoperative median of 21 (Figure 2). Postoperatively, all 4 studies found improvements in their scores. The weighted average of the 3 studies postoperatively was 70.5 ± 6.1, and the postoperative study that reported the median was 66. One study^3^ reported the VAS-FA score preoperatively and postoperatively and found a statistically significant improvement (*P* ≤ .05) from 27.78 ± 3.98 preoperatively to 70.76 ± 7.72 postoperatively.

## Discussion

The primary finding of this systematic review is that transportal minimally invasive TTC nailing of the ankle is an effective adjunct to prepare the tibiotalar and the subtalar joint that leads to low rates of infection, with 1 reported deep wound infection and 4 surgical site infections, and relatively low rates of major complications. Additionally, transportal tibiotalar and subtalar joint preparation leads to good rates of complete ankle fusion. As such, minimally invasive transportal techniques such as arthroscopy and arthroscopic-portal fluoroscopic joint preparation are viable and important adjuncts when fusing an ankle with a TTC nail.

One of the main proposed benefits of arthroscopic-associated preparation of the tibiotalar and subtalar joint, compared to open, is the minimal disruption of soft tissues. This is exceptionally important in patients who already have preexisting issues with wound healing. As expected, this review found that with these minimally invasive techniques, there were very few infectious complications associated with TTC nailing. In particular, there were 5 total incidences of infection (7.7% of ankles), with only 1 ankle having a deep infection and 1 ankle needing irrigation and debridement. One included study^2^ compared arthroscopic to open TTC arthrodesis in high-risk patients. It found excellent union rates for both arthroscopic and open TTC nailing in patients without preexisting ulceration. However, in their high-risk patient cohort (patients with significant comorbidities such as diabetes and peripheral arterial occlusive disease), 50% of open TTC fusions were complicated by surgical site infections, whereas 0% of arthroscopic TTC fusions in their studies resulted in major surgical site infections.^2^ This is incredibly important for patients with diabetes mellitus, as Wukich et al^28^ (117 open TTC arthrodesis) found an increase in the likelihood of surgical site infections by a factor of 8 in diabetic patients.^2^ In a cohort of 179 patients undergoing open TTC arthrodesis, there was an amputation rate of 12% and diabetes mellitus was the strongest risk factor for amputation, with an increased odds ratio of 7.^7^ Similarly, a study^2^ included in this review found the rate of amputation in open TTC fusion to be 13% compared to 0% in their arthroscopic cohort. In another recent retrospective review of 101 patients undergoing open TTC fusion with various approaches, they found an infection rate of 37% and 29.6% for patients with nontraumatic osteoarthritis and Charcot arthropathy, respectively.^18^ Additionally, 5 patients (4.9%) went on to require below-knee amputations.^18^ Although there may be significant heterogeneity when comparing patients undergoing open and minimally invasive TTC fusion, transportal TTC fusion should be considered in patients with significant comorbidities, particularly those with diabetes mellitus, as it leads to low rates of surgical site infections and thereby may reduce the risk of severe complications such as amputations.

Reducing complications in general for all patient populations is an important consideration when deciding whether to perform open or minimally invasive TTC fusion. The reported complication rates vary from 1% to 56% with open joint preparation–associated TTC nailing,^2,4,6,12,19,28^ which is comparable to the overall complication rate of 38.5% found in this study, although patient populations may be different. Infection remains one of the most common complications in open TTC fusion, regardless of the patient’s comorbidities.^9^ A previous 2016 systematic review examining^9^ 31 studies that reported complications after open TTC fusion found that the most common complications were infection and hardware irritation requiring removal. They found an overall infection rate of 11.9% after open TTC fusion compared to the overall rate of infection of 7.7% after minimally invasive joint preparation for TTC nailing found in this current review. However, in another series of 17 patients undergoing open TTC arthrodesis with headless compression screws, only 1 patient had poor surgical wound healing, with a complication rate of 5.9%.^10^ As such, transportal and open TTC nailing appear to have comparable rates of overall complications and low postsurgical infections in healthier patients.

The primary endpoint when using a TTC nail is to achieve a complete osseous hindfoot fusion. Regardless of the technique, without achieving full ankle fusion, the patient can experience residual pain and instability. The rates of full ankle fusion in open TTC arthrodesis range from 55% to 100%.^2,4,6,12,19,28^ One of the possible concerns with arthroscopic TTC fusion is the inability to properly prepare the tibiotalar and/or subtalar joint for fusion leading to pseudoarthrosis or nonunion. This review found that there was full ankle fusion in 86.2% ± 16.7% of patients (range: 57%-100%). In the previous systematic review of 31 studies evaluating open TTC arthrodesis, they found the rates of nonunion to be 7.5%.^9^ Similarly, they found the time to ankle fusion for open TTC arthrodesis to be 19.5 weeks, compared to 12.9 ± 2.0 weeks in the current systematic review. In the recent retrospective review of 101 patients that underwent open TTC fusion, they found 29 patients (28.7%) that went on to nonunion.^18^ Therefore, transportal TTC fusion may have comparable rates of full ankle fusion and time to fusion when compared to open TTC fusion; however, differences in patient populations should be examined further.

Minimally invasive transportal (using either an arthroscope or fluoroscopy) TTC nailing is a technically demanding procedure. This systematic review summarizes the techniques used in the included studies to provide examples and steps for those that may be less comfortable with minimally invasive tibiotalar and/or subtalar joint preparation. There were very comparable procedures between the studies, with 4 studies using an arthroscope to directly visualize the joint and 1 study using the same arthroscopic portals but using fluoroscopy to prepare the joint. Fluoroscopic joint preparation, without the use of an arthroscope, is a relatively new option for preparing a joint prior to fusion. The disadvantage of arthroscopic joint preparation is that the bony debris and bone slurry that is created by preparing the joint is removed because of the circulating arthroscopic fluid.^29^ This bony debris created may improve joint fusion rates; therefore, with fluoroscopic joint preparation there is the added benefit of minimally invasive surgery while retaining these bony by-products.^27^ Regardless of which technique was used to aid joint preparation, the key difference between open joint preparation, and what may attribute to the difference in outcomes, is the minimal disruption of soft tissues as the outcomes when using an arthroscopic and fluoroscopic guidance were very comparable. The key finding, again, is that by creating small incisions in the skin and soft tissues to access the joint, the surgeon is able to adequately prepare the joint(s), leading to good rates of ankle fusion as well as low rates of infections.

## Limitations

The greatest limitation of this study is that there are very few studies, and of lower level of evidence, that report the outcomes of patients undergoing minimally invasive transportal tibiotalar and/or subtalar joint preparation using an arthroscope or fluoroscopy prior to TTC ankle fusion, which reflects the novelty of this surgical technique. Nonetheless, this review suggests that surgeons should consider incorporating this technique into their armamentarium. Second, without the benefits of randomization and anonymization, this systematic review based mostly on case series can lead to confounding factors and biases that can impact the outcomes of the procedures. For example, we do not know if patient selection was biased toward less deformed or challenging problems for transportal approaches than for open approaches and thus confound generalizability of the review’s findings. Thus, when comparing patients who underwent transportal TTC fusion to open TTC fusion, there are limitations on the conclusions that can be made given the significant heterogeneity in patient populations.

## Conclusion

This systematic review presents an up-to-date summary of all the studies that have examined the somewhat limited initial studies reporting the complications and results of transportal TTC nailing. From this review, the authors believe that transportal joint preparation TTC nail ankle fusion is a reasonable approach that appears at least as safe and effective as open approaches for indicated patients.

## Appendix 1. Search Strategy

**Table:**

MEDLINE & EMBASE	Web of Science
1. tibiotalocalcan*.mp. 2. tib*.mp. 3. ankle.mp. or exp Ankle Joint/ or exp Ankle/ 4. fusion.mp. 5. arthrodesis.mp. or exp Arthrodesis/ 6. 2 or 3 7. 4 or 5 8. 6 and 7 9. exp Arthroscopy/ or arthros*.mp. 10. 8 and 9 11. 1 or 10	((ALL=(ankle) OR ALL=(TTC) OR ALL=(tibiotalocalcaneal)) AND ALL=(arthrosc*)) AND (ALL=(fusion) OR ALL=(Arthrodesis))

## Appendix 2. Patient Setup.^a^

**Table:**

Author	Positioning	Anesthesia	Tourniquet	Pump Pressure	Time of Ischemia (h)	Average Length of Procedure (min)	Traction	Adjuncts	Irrigation
Baumbach et al^2^	Supine	NR	NR	NR	NR	NR	NR	NR	NR
Biz et al^3^	Supine	Spinal Anesthesia	No	NA	NA	80.16 ± 8.12	No	No	NR
Guerra Álvarez et al^11^	Prone	Spinal Anesthesia	Yes	NR	2 (median)	NR	NR	NR	NR
Mencière et al^15^	Prone	NR	NR	NR	NR	NR	NR	NR	NR
Sekiya et al^22^	Supine	NR	NR	NR	NR	NR	NR	NR	NR

Abbreviation: NR, not reported.

a The characteristics and room setup when preparing the patients for surgery along with adjuncts used.

## Appendix 3. Intramedullary Nailing.^a^

**Table:**

Author	Intramedullary Rod	Nail Insertion Steps	Ankle Fusion Position
Baumbach et al^2^	1. Expert HAN (DePuy/Synthes, Oberdorf, CH) = 4 patients 2. AFN (Tornier, Montbonnot Sant Martin, France) = 11 patients	1. To determine entry point, lateral fluoroscopy views were obtained and a guidewire was placed in line with the tibial canal. 2. In the coronal plane, the guidewire was positioned at the lateral column of the calcaneus. Then, the guidewire was inserted through the center of the lateral column of the calcaneus up to the center of the talar dome just penetrating the tibia. 3. Then, drilling was performed to a depth of 1 cm beyond the tibial articular surface. 4. The 2.0-mm K-wires were removed, the hindfoot was placed in varus, and a reaming rod was inserted. 5. Reaming of the distal tibia was performed until the reamer demonstrated good bony contact. 6. Nail diameter was determined according to the reaming. 7. The nail was inserted and locked using the targeting jig.	NR
Biz et al^3^	1. Retrograde intramedullary titanium (Ti-6AI-4V) nail (Panta Nail, LifeSciences).	1. Ankle alignment was restored before application of the nail. 2. A 2.5-cm plantar incision located at the intersection point of the line passing from the second toe to the calcaneal center and the bimalleolar line 3. A 3.2-mm K-wire with a length of 400 mm was inserted through the calcaneus and talus under fluoroscopic guidance until it reached the tibial intramedullary cavity. 4. The subtalar and tibiotalar articular surfaces were reamed over the guide pin. Reaming continued sequentially until the final reaming of 0.5 mm larger than the implant. 5. K-wire was removed and the intramedullary nail was inserted. A K-wire was inserted through the tibia to support the nail in the correct position. 6. The nail was fixed distally with 2 distal locking screws to the calcaneus. 7. A compression device was used. The proximal tibial K-wire was removed and the guides for the 5-mm reamers were positioned. Holes were drilled depending on final implant length and compression rods were inserted. Then compression was started to a maximum of 12 mm. 8. After compression, the intramedullary nail was fixed to the bone with two 3.5-mm screws. 9. An end cap was used on the plantar hole of the nail.	Axial: 10-15 degrees of external rotation Coronal: 5 degrees of valgus
Guerra Álvarez et al^11^	NR	The locked locking nail is inserted via the plantar route	Sagittal: Neutral Axial: 5 degrees external rotation Coronal: 0-5 degrees hindfoot valgus
Mencière et al^15^	1. T2 system (Stryker Orthopaedics, Kalamazoo, MI)	1. Plantar approach was made along the axis of the fourth metatarsal, just in front of the heel-ground contact point 2. The guidewire was placed into the tibial shaft under X-ray guidance 3. The bone was reamed progressively 4. The nail was introduced and locked dynamically. No distractors were used.	NR
Sekiya et al^22^	1. Intramedullary nail with fin (Nakajima Propeller Inc, Okayama, Japan) IMN: 8 mm diameter, 180 mm length	1. A 3-cm transverse incision was made on the plantar aspect of the foot under the calcaneus. 2. A guidewire was passed through the calcaneus and talus into the tibia under fluoroscopic guidance. 3. Reaming was performed. 4. The intramedullary nail was inserted over the guidewire 5. The distal end of the nail was driven 5 mm inside the calcaneus. 6. Two proximal fixation screws were inserted from the medial side	Sagittal: Neutral axial: 5-degree external rotation Coronal: 5-degree valgus

Abbreviation: NR, not reported.

a Detailed steps for inserting and fusion the ankle using a tibiotalocalcaneal nail.

## Appendix 4. Postoperative Protocol.^a^

**Table:**

Author	Weightbearing
Baumbach et al^2^	Nonweightbearing in a cast for 8 wk. CT scans were conducted 8 wk postoperatively and every 6 wk after until bony fusion was detected
Biz et al^3^	Nonweightbearing in a cast for 4 wk, Then partial weightbearing for 6 wk
Guerra Álvarez et al^11^	Nonweightbearing first 4 wk in a splint Transitioned to nonarticulated Walker-type boot on the fourth week after surgery—now able to partially weightbear with crutches Orthosis is removed at 12 wk: no full weightbearing without Walker-type boot until radiographic confirmation of bone consolidation.
Mencière et al^15^	NR
Sekiya et al^22^	Variable: average period until full weightbearing was 7 wk (range 2-11 wk)

a The details of the postoperative protocol as defined in each article.

Abbreviation: NR, not reported.
